# Impact of Omega-3 and Omega-9 fatty acids enriched total parenteral nutrition on blood chemistry and inflammatory markers in septic patients

**Published:** 2014

**Authors:** Gamze Gultekin, Habibe Sahin, Neriman Inanc, Fatma Uyanik, Engin Ok

**Affiliations:** 1 Gamze Gultekin, MSc, Formortaca Diet Clinic, Mugla, Turkey.; 2 Habibe Sahin, Associate Professor, Department of Nutrition and Dietetics,; 3 Neriman Inanc, Professor, Department of Nutrition and Dietetics,; 4 Nuh Naci Yazgan University, Faculty of Health Sciences, Kayseri, Turkey.; 5 Fatma Uyanik, Professor, Canakkale Onsekiz Mart University,; 6 Health Services Vocational College, 17000, Canakkale, Turkey.; 7 Engin Ok, Professor, Department of General Surgery, Erciyes University, Faculty of Medicine, 38039, Kayseri, Turkey.

**Keywords:** Biochemical parameters, Inflammatory markers, Olive oil, Sepsis, TPN

## Abstract

***Objective:*** Lipid emulsions containing omega-3 are known to have positive effects on patient's prognosis due to anti-inflammatory properties. The aim of this study was to investigate the effects of omega-3 enriched total parenteral nutrition (TPN) emulsion containing omega-9 on biochemical parameters, inflammatory mediators in septic patients.

***Methods:*** Thirty-two participants who were not fed orally for over five days and needing TPN support were included in this prospective, randomized and double-blind clinical study. Patients were randomly divided into control (n=16), treatment (n=16) groups. The treatment group received TPN containing 80% olive oil+20% soy oil additionally 10 g fish oil enriched TPN. Control group received only olive oil containing standard lipid emulsion (1.3±0.1 g/kg/day). Blood samples were collected for biochemical analysis on the 1^st^ and 6^th^ days of study.

***Results:*** The serum albumin levels significantly increased (*p*<0.05) in both groups whereas total protein and prealbumin levels did not show any significant changes. In treatment group, significant decreases were determined in LTB_4_ and CRP levels (*p*<0.05) while decreases in IL-6, TNF-α and leukocyte levels were not significant. No statistically significant changes were found in LTB_4_, CRP, IL-6, TNF-α and leukocyte levels of controls.

***Conclusion:*** Results of the study have shown that omega-3 enriched TPN solution containing omega-9 contributes to decrease in the levels of inflammatory mediators and to improvement in the biochemical parameters in septic patients.

## INTRODUCTION

Sepsis, a systemic response of the host against infections is a complex syndrome that can lead to multiple organ failure, death.^1^ Recently, although pathophysiology of sepsis is better understood, advances is gained in diagnostic methods, technology, antimicrobial therapy, it still results in high mortality when complicated with shock and multi-organ system failure (MOSF). Sepsis is main cause of death in non-coronary intensive care unit (ICU).^2^ In ICU, sepsis causes increases in morbidity, mortality, expenses. Therefore, new treatment modalities for key positions are developed to reduce problems.^2^^,^^3^

Lipid emulsions used in parenteral nutrition are generally soy oil rich in omega-6 fatty acids. Recently, emulsions containing fish oil rich in omega-3 fatty acids have been developed due to their positive effects on immune system.^4^ Immunomodulatory effects and therapeutic benefits of omega-3 fatty acids have been shown in different diseases in clinical and experimental studies. Infusion of omega-3 was reported to reduce serum tumor necrosis factor-α (TNF-α), interleukin-10 (IL-10) levels, increase nitrogen retention.^4^^,^^5^ Omega-3 fatty acids are thought to prevent/diminish hyper-inflammatory processes through signal cycle from cell to cell. They exert these effects by inhibiting pro-inflammatory arachidonic acid (AA) metabolites, release of platelet-activating factor (PAF), increasing formation of anti-inflammatory eicosapentaenoic acid (EPA) derivatives.^6^^,^^7^ In most studies, omega-3 fatty acid addition to diets reduced production of prostaglandin E_2_ (PGE_2_), thromboxane B_2_ (TXB_2_), LTE_4_ and LTB_4_ from inflammatory cells.^6^^-^^8^ In most studies investigating effects of olive oil-based lipid emulsion on immune cell functions, effects of olive oil-based lipid emulsions were found to be neutral compared to soy-based lipid emulsions.^9^^,^^10^ Emulsions containing omega-9 may exert indirect effects due to combining with omega-6 and omega-3 of membrane phospholipids.^9^

In critical conditions like sepsis, trauma, multiple organ failure, it is known that lipid emulsions containing omega-3 has positive effects on patient's prognosis due to anti-inflammatory properties. Olive oil-based lipid emulsions may also contribute to this effect. Therefore, this study was planned to investigate impact of omega-3 enriched TPN solution containing omega-9 on biochemical and inflammatory markers in septic patients.

## METHODS

***Patients and Study Design: ***The study procedures were arranged according to Helsinki Declaration and approved by local Ethics Committee. Study was performed in General Surgery Intensive Care Unit of Gevher Nesibe Hospital, Kayseri, Turkey. Fifty eight patients were recruited, 19 died, 7 were excluded due to non-application of TPN for 5 full days. Study was completed with 32 patients with mean age of 62.9±12.2 (min 44.0 - max 77.0) years.

Patients who were 18 years old or older and diagnosed with severe sepsis or septic shock having no ability to orally consume food for more than five days thus requiring TPN support were included in this prospective, randomized, double-blind clinical study regardless of primary diagnosis; pregnant patients were excluded from the study. Sepsis was diagnosed by physician according to criteria set by Critical Care Consensus Conference of Intensive Care Society of the Union of the American Physicians.^11^

***Nutritional Regimen: ***Patients were randomly divided into two groups. Oil emulsions being 30-40% of total energy were given to both groups. Treatment group received a mixture consisting 100 ml of omega-3 fatty acids (10% emulsion of Omegaven-Fresenius-Kabi containing 10 g fat, 1.25-2.82 g EPA, 1:44 -3:09 g DHA) and rest of the calculated total amount of oil was olive oil emulsion (20% emulsion of Clinoleic-Baxter containing 80% olive oil, 20% soy oil). Control group received only olive oil containing standard lipid emulsion. Emulsions were put into TPN bags with a compounder by a nurse. Researchers were blind to which oil emulsion was put into the bag; it was only known to the nurse.

Nutritional support was continued for at least 5 days in both groups. Basal energy requirements were calculated with the Schofield equation. Stress factors were calculated with the Scrimshaw formula, protein requirement was calculated according to non-protein energy/nitrogen ratio.^12^ Dextrose (50%), Dextrose (30%), Aminoplasmal (10%), HepatAmin (8%) (Baxter), NephrAmin (5.4%) (Baxter) were used as TPN solution. One vial of trace elements (Tracutil-Braun), 1 vial of fat-soluble and water-soluble vitamins (Cernevit-Baxter) were administered to each patient daily. Potassium, sodium, chlorine were arranged according to patient’s biochemical indicators. The TPN solutions were similar except for omega-3 fatty acids.

***Biochemical Measurements: ***Blood samples were collected from the vein in the mornings of the 1^st^ and 6^th^ days of the study by nurse in charge. Sera were collected after one hour incubation at room temperature, plasmas were separated immediately by centrifugation, samples were stored at -80^o^C until analysis.

Blood glucose, blood urea nitrogen (BUN), creatinine, bilirubin, total protein, albumin, blood lipid [total cholesterol, triglycerides, high density lipoprotein (HDL), low density lipoprotein (LDL)] levels and aspartate transaminase (AST), alanine transaminase (ALT) activities were determined by autoanalyzer (MEGA, Merck). Very low density lipoprotein (VLDL) and LDL levels were calculated with standard formulas (LDL cholesterol=Total cholesterol-VLDL cholesterol-HDL cholesterol; VLDL cholesterol=Triglycerides/5). C-reactive protein (CRP) levels were determined with nephelometric method using N High Sensitivity CRP kit on Dade Behring-2; prealbumin levels were detected with spectrophotometric method using Beckman-Coulter kit on IMAGE SYSTEM. Inflammatory mediators (TNF-α, IL-6, LTB_4_) were determined with commercial kits (Pierce Biotechnology, endogenous human TNF-α; Pierce Biotechnology, endogenous human IL-6, Rockford, USA and Thermo Scientific LTB_4_, Waltham, USA) by ELISA.

***Statistical Analysis: ***Data were analyzed by SPSS (version 15.0) software. For each variable, normal distribution curve was tested by Kolmogorov-Smirnov test. Comparisons of pre and post-treatment results were performed with Paired Samples t test. Data were expressed as means±SEMs. Statistical significance was set at p<0.05.

## RESULTS

***Energy and Nutrient Intake:*** Daily energy and macro nutrients intake of groups were not different (p>0.05) (Table-I).

**Table-I:** Energy and macro nutrient intakes of patients

*Variables*	*Treatment group (n:16)*	*Control Group (n:16)*	*p-value*
Age	62.0 ± 10.6	63.8 ± 13.7	NS
Male/Female	12/4	10/6	
Energy (kcal/kg/day)	27.5 ± 1.5	25.8 ± 1.5	NS
Carbohydrate (g/kg/day)	3.6 ± 0.2	3.4 ± 0.8	NS
Fat (g/kg/day)	1.3 ± 0.1	1.3 ± 0.1	NS
Protein (g/kg/day)	1.1 ± 0.1	1.3 ± 0.2	NS

**Table-II:** Effects of oil emulsions on serum proteins, lipids and inflammatory markers

*Characteristics*	*Treatment group*				*Control Group*			
	*Baseline*	*Final*	*t*	*p*	*Baseline*	*Final*	*t*	*p*
Protein (mg/dL)	5.1±0.3	5.2±0.3	0.422	NS	4.3±0.3	4.6±0.3	2.144	<0.05
Albumin (g/dL)	1.8±0.1	2.1±0.2	2.126	<0.05	1.5±0.1	1.7±0.1	2.78	<0.05
Prealbumin (mg/ dL)	9.7±1.0	9.5±1.1	0.753	NS	7.6±0.9	7.9±1.1	0.604	NS
Triglycerides (mg/ dL)	169.9±36.5	132.12±15.5	1.295	NS	232.5±40.4	240.7±55.7	0.356	NS
T.Cholesterol (mg/dl)	90.8±8.1	82.3±6.0	1.400	NS	106.4±11.95	104.6±11.4	0.209	NS
HDL-Cholesterol (mg/dL)	8.8±0.9	7.00±0.8	1.811	NS	15.1±1.6	11±1.2	2.942	<0.05
LDL-Cholesterol (mg/dL)	52.8±4.9	47.8±5.6	1.148	NS	60.1±17.5	47.8±7.5	0.665	NS
VLDL-Cholesterol (mg/dL)	34±7.3	26.7±3.3	1.267	NS	46.5±8.1	48.1±11.1	0.356	NS
LTB_4 _(pg/ml)	4653.9±1018.3	4061.3±1147.9	0.696	<0.05	1233.1±518.7	1433.6±415.8	0.862	<0.05
IL-6 (pg/ml)	54.2±12.4	36.4±5.6	1.623	NS	167.0±33.0	84.7± 25.3	1.410	NS
TNF-α (pg/ml)	22.7±2.6	22.1± 2.4	0.193	NS	37.9±5.5	32.2±3.7	1.403	NS
CRP (mg/dL)	129.4±10.9	103.0±9.5	2.316	<0.05	183.3±27.4	168.1±20.2	1.164	NS
Leukocyte (10^9 ^L)	17.2±2.2	14.0±1.4	1.609	>0.05	15.1±1.1	17.1±1.2	1.681	>0.05

**Table-III:** Distribution of the patients according to the duration of hospitalization, receiving TPN and returning to enteral nutrition

*Duration (day)*	*Treatment group*	*Control Group*	*p-value*
Stay in hospital	31.6 ± 4.3 (12-60)	30.6 ± 4.3 (7-67)	NS
Receiving TPN	23.7 ± 3.8 (6-52)	19.1 ± 4.0 (5-62)	NS
Returning to Enteral Nutrition	5/16	7/16	

***Serum Proteins: ***Total protein and prealbumin levels increased compared to pretreatment in both groups being significant (p<0.05) in controls, but not in the treatment group. Post-treatment albumin levels were significantly increased in both groups (p<0.05) (Table-II).

***Serum Lipids: ***In treatment group, decreases in triglycerides, total cholesterol, LDL, VLDL and HDL-cholesterol levels were not significant (p>0.05). Slight but not significant increases were determined in triglyceride and VLDL-cholesterol levels, also slight reductions were found in total and LDL-cholesterol levels. HDL-cholesterol levels decreased significantly (p<0.05) in controls (Table-II).

***Inflammatory Markers: ***No significant differences were detected between pre- and post-treatment IL-6 and TNF-α levels in both groups (p>0.05). At the end, reductions observed in CRP levels of both groups being significant only in treatment group (p<0.05). LTB_4_ levels increased in controls but decreased in the treatment group (p<0.05) (Table-II).

***Prognosis: ***Only 12 patients (37.5%) could return to enteral nutrition. Totally 17 patients (53.1%) (9/16 and 8/16) survived, being statistically insignificant (p>0.05). Duration of hospitalization and TPN support were 31.6±4.3 days and 23.7±3.8 days for treatment group and 30.6±4.3 days and 19.1±4.0 days for control group, respectively (p>0.05) (Table-III).

## DISCUSSION

Sepsis, a complex syndrome, can lead to multi-organ failure and death resulting in high mortality rates in many non-coronary ICUs.^2^^,^^3^ Among new treatment approaches, lipid emulsions have gained importance due to being energy sources, anti-inflammatory and pharmacological effects.^4^ Although common soy oil-based lipid emulsions have pro-inflammatory potential due to high content of omega-6 fatty acids, their immunological effects are not so clear. Omega-3 fatty acids different from omega-6 in biological activity showed beneficial effects on cellular defense and inflammation.^5^

***Serum Proteins: ***Albumin, transferrin, total protein, prealbumin levels reflect visceral protein stores.

To follow up short term nutritional treatments, prealbumin having a half-life of 2 days is accepted as a good indicator to assess the responses to nutritional treatment.^13^^,^^14^ Therefore, prealbumin levels were also evaluated; prealbumin levels declined in the treatment and increased in the control groups at the end of the study being statistically insignificant (Table-II). In critical patients in ICU, serum albumin levels may be diminished not only due to stress of illness, surgery, trauma, sepsis, increased peripheral destruction but also to liver-kidney dysfunctions which may reduce albumin levels.^14^^,^^15^ Hypoalbuminemia develops rapidly due to extravasations of albumin in septic patients. Depending on increased IL-1 synthesis, changes may occur in protein synthesis in favor of the synthesis of acute phase proteins instead of albumin in the liver, which also accelerates severity of hypoalbuminemia.^13^ In this study, lower albumin levels which might be associated with catabolism increased significantly in both groups (Table-II).

***Serum Lipids: ***Relationship between systemic inflammation and lipid metabolism has been shown in clinical trials. It has been reported that insulin resistance and cytokines such as TNF-α, IL-1, IL-2, IL-6, PAF raise serum triglyceride levels while reduce total and HDL-cholesterol levels.^16^

In this study, serum triglyceride levels decreased in the group receiving omega-3 at the end of the study compared to pre-treatment whereas an increase was observed in the group receiving omega-9 (p>0.05). In control and treatment groups, determination of decreases in total, LDL and VLDL-cholesterol levels was consistent with results of previous studies but not reached to statistical significance (Table-II).

***Inflammatory Markers: ***Soy oil based lipid emulsions commonly used in TPN are rich in PUFA, particularly, in linoleic acid (LA). Intake of excessive LA impairs synthesis of long-chain PUFA by acting on desaturation and elongation steps, leads to imbalances in the synthesis of eicosanoids and suppresses immunity. Moreover, suppressive effect of omega-6 fatty acids on immune function has been shown by many studies.^4^^-^^6^

Recent studies have been focused on immunological effects of olive oil emulsions. In an in vitro study, Reimund et al^17^ have shown that olive oil-based emulsions reduced synthesis of TNF-α and IL-1 in mononuclear cells. Olive oil emulsion slightly suppressed lymphocyte proliferation and IL-2 production.^18^ In most studies investigating effects of olive oil-based emulsions on immune cell functions, effects of olive oil emulsions were more neutral than soy-based lipid emulsions.^18^^,^^19^

One of the omega-3 fatty acids, EPA, which is precursor of synthesis of biologically highly active eicosanoids influences inflammatory reactions.^5^^,^^20^ In many studies, omega-3 fatty acids supplementation to diet was shown to reduce the release of PGE_2_, TXB_2_, LTB_4_, LTE_4 _from inflammatory cells.^8^^,^^11^ These results suggest that omega-3 fatty acids can be used as potential anti-inflammatory agents in septic patients or patients at risk of hyper-inflammatory response.^4^^,^^5^^,^^21^

The CRP is the most well known acute phase protein. Levels of CRP remain high as long as inflammation and tissue damage continue. Half-life of CRP is 4-6 hours, therefore it returns to normal levels within 3-7 days after termination of the inflammation.^2^ Grecu et al^22^ found significant decreases in serum CRP concentrations in 88% of septic patients receiving lipid emulsion consisting fish and soy oils at the rates of 66% and 33%, respectively for over 5 days. The CRP levels of treatment group decreased (from 129.4±10.9 mg/dL to 103.0±9.5 mg/dL) and this significant (p<0.05) reduction was consistent with literature. Decrease in controls (from 183.3±27.4 to 168.1±20.2 mg/dL) was not significant in the present study (Table-II). Although TNF-α is the most important mediator of inflammatory process in sepsis, it is only one of the cytokines involved in septic response. There are several studies showing increased IL-6 concentrations in patients died from sepsis.^2^^,^^16^^,^^23^ In this study, TNF-α levels increased slightly in treatment group whereas slightly decreased in controls (p>0.05) (Table-II). Baseline TNF-α levels were not important in determining prognosis of septic patients, TNF-α levels showed different profiles depending on progression of the disease and remained high in cases resulting in death.^16^

In animal models, it has been shown that productions of TNF-α and IL-6 in macrophages^1^^,^^23^ and their serum levels^17^ decreased in omega-3 fatty acids administrated septic rats. Levels of IL-6, one of the inflammatory markers, decreased in both groups at the end of the study compared to pre-treatment. Despite being insignificant (p>0.05), this decrease confirmed the results of previous studies.^24^^,^^25^

Omega-3 fatty acids increase the synthesis of pro-inflammatory LTB_5_ which is less active than LTB_4_.^4^ Many studies using different levels of fish oil have proven that omega-3 fatty acids reduced LTB_4_ levels.^4^^,^^7^^,^^8^^,^^22^ Morlion et al^15^ have shown that parenteral fish oil application to postoperative patients for 5 days increased LTB_5_/LTB_4_ several folds and exerted immunomodulatory effect on leukocytes. Consistent with Morlion et al^15^, significant reduction was found in LTB_4_ levels of treatment group in contrast to the increases in controls in this study (p<0.05). Leukocytosis or leukopenia can be seen in complete blood counts of septic patients. Leukocytosis is developed through release of neutrophils from bone marrow to peripheral circulation by the effect of cytokine, neutrophils migrate to inflammation site by the effect of IL-8.^2^ Leukocyte count is usually above 12.000/mm^3^.^3^^,^^5^ There is a well-known relationship between increased number of peripheral white blood cell and acute responses to infection, trauma or inflammation. This is considered as part of acute phase responses. In this study, occurrence of leukocytosis was high in patients in both groups, which is the consequence of normal course of sepsis. High leukocytosis level decreased in the treatment group, but it increased in controls (p>0.05).

It is clear that nutrition is the most important supportive therapy due to its positive effects on the results and improvement in patient's condition. Results of this study have shown that omega-3 fatty acids can be used safely for contribution of metabolic responses in septic patients due to their effects on inflammatory markers (CRP, LTB_4_, TNF-α). However, further studies are required to highlight the effects of lipid emulsions on inflammatory responses in septic patients.

## Authors Contribution:

**GG, HS, NI:** Study design.

**GG, HS, FU: **Data collection and analysis.

**NI, FU, EO: **Manuscript preparation.
